# Metastatic Clear Renal Cell Carcinoma in Urinary Bladder at Presentation—A Rare Incidental Case Report and Literature Review

**DOI:** 10.1002/ccr3.70199

**Published:** 2025-02-10

**Authors:** Rao Nouman Ali, Adeel Anwaar, Wajiha Irfan, Farooq Hameed, Inam Ul Haq, Muhammad Asif Raza, Riyan Imtiaz Karamat, Aymar Akilimali

**Affiliations:** ^1^ District Head Quarter Hospital Khanewal Pakistan; ^2^ Punjab Rangers Teaching Hospital Lahore Pakistan; ^3^ Urology Suite Midcity Hospital Lahore Pakistan; ^4^ Combined Military Hospital Multan Multan Pakistan; ^5^ Hijaz Hospital Gulberg Lahore Pakistan; ^6^ Rashid Latif Medical and Dental College Lahore Pakistan; ^7^ RHQ Hospital Skardu Skardu Pakistan; ^8^ Rahbar Medical and Dental College Lahore Pakistan; ^9^ Department of Research Medical Research Circle (MedReC) Goma Democratic Republic of Congo

**Keywords:** bladder carcinoma, case report, clear cell carcinoma, gross hematuria

## Abstract

This case highlights the rare occurrence of concurrent clear cell renal carcinoma with metastatic urinary bladder involvement, presenting as hematuria and flank pain. Initial screening revealed both renal and bladder tumors, confirmed as metastases. Histopathology emphasized thorough investigations for unexplained urinary symptoms and the need for multimodal treatment and follow‐up.

**Abbreviations**
ccRCC: clear cell subtype of renal cell carcinoma
IMDC: international metastatic renal ca database consortium
Met RCC: metastatic renal cell carcinoma
MSKCC: memorial sloan kettering cancer center
OS: overall survival
PFS: progression‐free survival
RCC: renal cell carcinoma
TKI: tyrosine kinase inhibitors
TURBT: transurethral resection of bladder tumor
VHL syndrome: von hippel lindau syndrome

## 1 Introduction

Renal cell cancer (RCC) is well‐known worldwide for its insidious pattern of aggressiveness and late presentation. RCC accounts for 2%–3% of all new cancers detected annually, with a higher incidence in males at a ratio of 2:1. About 15%–20% of RCC patients present with metastasis at the time of diagnosis worldwide. Geographically, the incidence is higher in the Western world and the United States compared to Asia, being approximately 15 times more common [1]. RCC is the 6th most frequent cancer in males and the 10th in females worldwide, and according to the WHO, over 140,000 people die each year due to RCC [2].

The most common presentation of renal cell cancer is gross hematuria with clots and a painful flank mass. This cancer has many subtypes, of which the most common is clear cell carcinoma, accounting for about 60%–80% of cases [3]. Renal cell cancer tends to metastasize before presenting with any local signs or symptoms. The most common sites of metastasis are the lungs, liver, bones, and brain, and in about 30% of patients, this cancer presents with the simultaneous presence of the primary tumor and metastatic deposits radiologically. Several rare sites of metastasis have been documented in the literature, including ureteric stumps, the prostatic fossa, retroperitoneal space, and urinary bladder. Metastasis of renal cell cancer to the urinary bladder has an incidence of 1%–3%, and in many cases, the tumor is diagnosed within 6 months of radical nephrectomy or after 6 months, as many such synchronous or metachronous tumor cases have already been reported in the literature [4]. The cases of synchronous and metachronous appearance of bladder metastasis after removal of the primary tumor are challenging to manage because of the diversity of the tumor nature, the difference in presenting complaints, the diverse need for treatment options, and the quantification of respective risk factors (Table 1 [5, 6, 7, 8, 9, 10, 11, 12]). Many cases are also reported in the literature with the presence of different types of tumors at a time, like renal cell cancer in the kidney along with transitional cell carcinoma in the ureter or bladder [5].

**TABLE 1:** literature review with comparison of previous published cases.

Journal	Author	Age yrs	Type	Sites of metastasis	Time taken by metastasis to appear in urinary bladder	Size and location of bladder metastasis	Symptoms	Past history	Management of metastasis	I&H (immune‐histo‐chemical) staining
Oncology letters	Hengping li et al. [5]	70	Synchronous	Urinary Bladder + retroperitoneal space	After 1 month	1.1 × 1.4 cm on left wall of bladder	Gross hematuria	Left open radical nephrectomy	TURBT + systematic therapy not given due to weak status of pt	Positive for vimentin, CD 10, PAX 2 Negative for prosaposin PSA, Uroplakin 3
Case reports in urology	Amanda smart et al. [6]	69	Metachronous	Only urinary bladder	After 8 months	5 cm lesion arising from left ureteric orifice	Gross hematuria	Laparoscopic left radical Nephrectomy	TURBT initially Left ureterotomy + partial cystectomy later	PAX 8 positive GATA 3 negative
International journal of surgical case reports	Mahdy alief adhiguna et al. [7]	62	Metachronous	Bladder + brain	After 4 years	2.5 × 2.5 anterior bladder wall	Gross hematuria + infrequent seizures	Left radical nephrectomy	TURBT + mastectomy for extra axial mass in parietal region	Not explained
BMC urology	Mustafa babar et al. [8]	79	Metachronous	Urinary Bladder, left acetabulum, left rib, lungs, thyroid, right renal vein, IVC	After 28 years	3.7 × 3.2 mass on right wall of bladder	Gross hematuria	Left sided radical nephrectomy	TURBT Chemotherapy Sunitinib initially later nivolumab	Not mentioned
Journal of laboratory physicians	Saha arpita et al. [9]	55	Metachronous	Only Urinary bladder	After 2 years	3 × 4cm ant wall and dome of bladder	Gross hematuria	Lap right radical nephrectomy	TURBT	Positive for CD10, vimentin AE 1, AE 33 Negative for EMA, CK 7, CK 20
Urology case reports	Dennisn boynton et al. [10]	64	Metachronous	Urinary Bladder, omentum	After 20 months	Near left ureteric orifice	Gross hematuria + abdominal discomfort	Left robotic partial nephrectomy	TURBT + laparoscopic excision of omental mass	Not mentioned
Asco publications	Alexander chehrazi‐raffle et al. [11]	83	Metachronous	Urinary Bladder + pulmonary	After 14 months	3.7 × 1.4 cm right bladder wall	Gross hematuria + bronchitis	Left robot Assisted Cytoreductive nephrectomy	TURBT + Systematic therapy	Positive for PAX 8, PAX 2, CA‐9 & Carbonic anhydrase
Journal of surgical casereports	Loannis zachos et al. [12]	85	Metachronous	Urinary Bladder + thorax	More than 6 months	30 mm near right ureteric orifice	Gross hematuria	Left radical nephrectomy	TURBT + immunotherapy for thorax metastasis	Positive for AE1/AE3, cyst 7, GATTA3, P63, PAX 8, vimentin and EMA, keratins Negative for cyst 20 & CD 10
Therapeutic advances in urology	Ruben de groote et al. [13]	80	Metachronous	Urinary bladder + liver	After 5 years	3 cm from right ureteric orifice	Gross hematuria	Left radical nephrectomy	TURBT Liquefication of liver metastasis under cover of systematic therapy	GATA 3 negative

This case is notable for the simultaneous presence of a mass in the left kidney and a separate polypoid growth in the urinary bladder. The patient underwent a transurethral resection of the bladder tumor (TURBT), followed by a left radical nephrectomy within the same surgical setting, with both tumor samples sent for histopathological analysis. The histopathology report confirmed that both the kidney and bladder tumors were of the clear cell carcinoma subtype of renal cell carcinoma.

## 2 Case Presentation

### 2.1 Case History/Examination

A 58‐year‐old male patient presented with a 3‐month history of intermittent flank pain and hematuria, characterized by the presence of thread‐like clots. There were no symptoms of headache, vomiting, palpitations, ataxia, vision loss, or excessive sweating; hence, no associated symptoms of VHL syndrome were found, along with a normal fundoscopic examination of the eyes. He has a medical history of hypertension and Type 2 diabetes mellitus, both of which have been managed with regular medications for the past 7 years, utilizing metformin 500 mg twice a day and losartan potassium 50 mg once a day with good compliance. Upon physical examination, a palpable mass was detected on the left side of the abdomen, accompanied by mild tenderness upon deep palpation. Laboratory investigations revealed largely unremarkable results, with the exception of a hemoglobin level of 10 g/dL, indicating anemia (normal range 13–17 g/dL), and a serum creatinine level of 1.7 mg/dL (normal range 0.7–1.3 mg/dL), suggestive of mild renal impairment.

### 2.2 Imaging Investigation Findings

**FIGURE 1:**
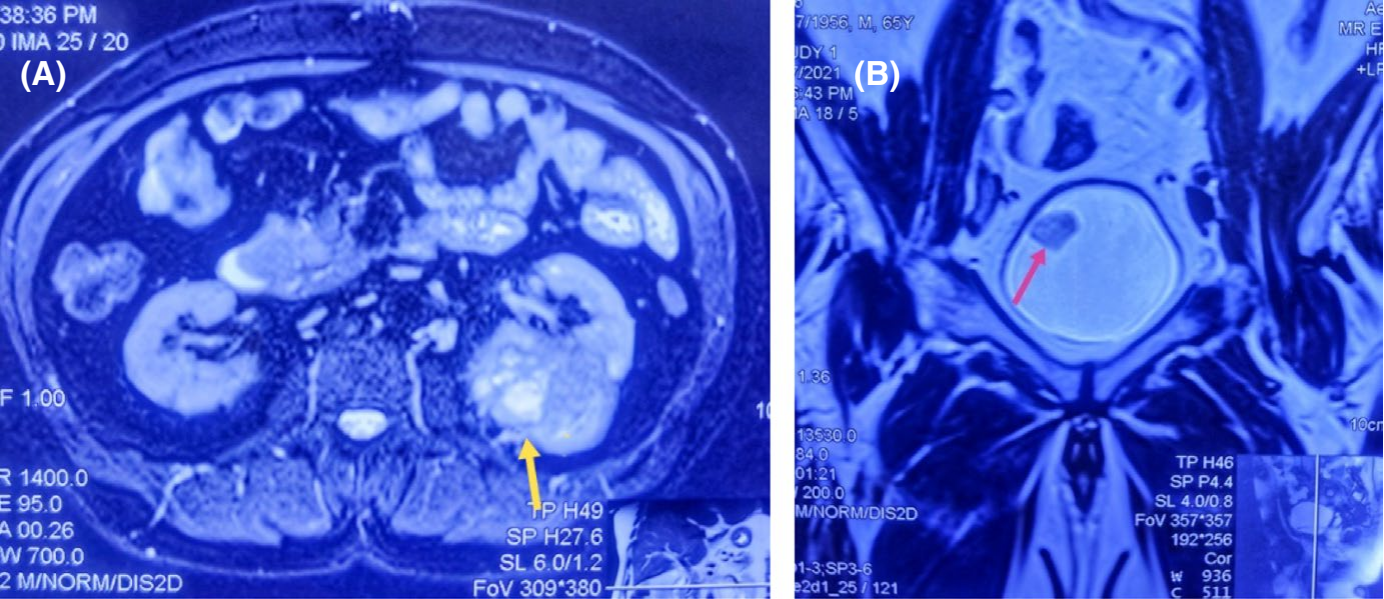
(A) Contrast‐enhanced MRI abdomen and pelvis (axial section) shows significantly enhanced left renal mass about 7 × 7.4 cm with mixed T1/T2 signal intensity (yellow arrow). (B) Contrast‐enhanced MRI pelvis (Coronal section) shows T2signal intensity soft tissue pedunculated lesion about 2 × 2.5 cm along the right lateral wall of the urinary bladder (pink arrow).

Ultrasonography revealed a heterogeneous tumor in the left kidney, characterized by a solid component measuring 5.5 × 4.8 × 6.0 cm. This mass was located at the upper, middle, and part of the lower pole of the left kidney. Additionally, a similar echogenic lesion measuring 2.0 × 2.5 cm was incidentally discovered on the right lateral wall of the urinary bladder. Subsequent abdominopelvic computed tomography (CT) and MRI confirmed the presence of an ill‐defined contrast‐enhanced tumor in the middle and lower poles of the left kidney, measuring about 7 × 7.4 cm (Figure 1A). Notably, there was no involvement of the renal vein or inferior vena cava, and no evidence of regional lymphatic metastasis was observed. Furthermore, the MRI of the pelvis identified a contrast‐enhanced lesion in the urinary bladder, measuring approximately 2.5 × 2.5 cm, located on the right lateral wall (Figure 1B). Importantly, there were no radiological signs of metastasis to any other organs. On MRI of the brain, no hemangioblastoma was detected, and no pancreatic endocrine tumor or pheochromocytoma was detected on contrast‐enhanced CT of the abdomen and pelvis. Genetic testing for various genes, including VHL, was found to be unremarkable.

### 2.3 Surgical Management

**FIGURE 2:**
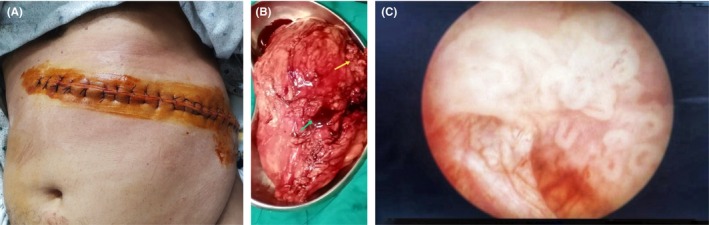
(A) An anterior left subcostal incision is utilized for left radical nephrectomy. (B) Left renal mass of about 7 × 7 cm irregular in shape, non‐capsulated, reddish‐brown in color with areas of hemorrhage and mild infiltration of perinephric tissue (yellow arrow) and renal pelvis (green arrow). (C) An endoscopic view of the bladder tumor shows 2 × 2.5 cm solitary, pedunculated growth along the right lateral wall of urinary bladder.

After comprehensive optimization of the patient's condition, a transurethral resection of the bladder tumor and an open radical nephrectomy were performed. During the procedure, the bladder tumor was identified as a broad‐based solitary lesion located on the right lateral wall, which was successfully excised in its entirety. The renal tumor was noted to be solid, ill‐defined, exerting pressure on adjacent structures; yet there was no involvement of the renal vein or inferior vena cava. The mass was irregular in shape, not well‐defined, non‐capsulated, reddish‐brown in color with areas of hemorrhage, and non‐hydronephrotic in appearance. Both resected specimens were submitted for histopathological examination (Figure 2A–C). After the nephrectomy, the patient was prescribed the calcium channel blocker amlodipine 10 mg for hypertension management, with the advice of regular blood pressure checkups and a decrease in dietary protein to < 1 g/kg/day, and sodium intake was reduced to 4 g/day. He was also advised to have monthly investigations of serum electrolytes, renal function tests, uric acid levels, and fasting lipid profile on a regular basis.

### 2.4 Histopathological Findings

**FIGURE 3:**
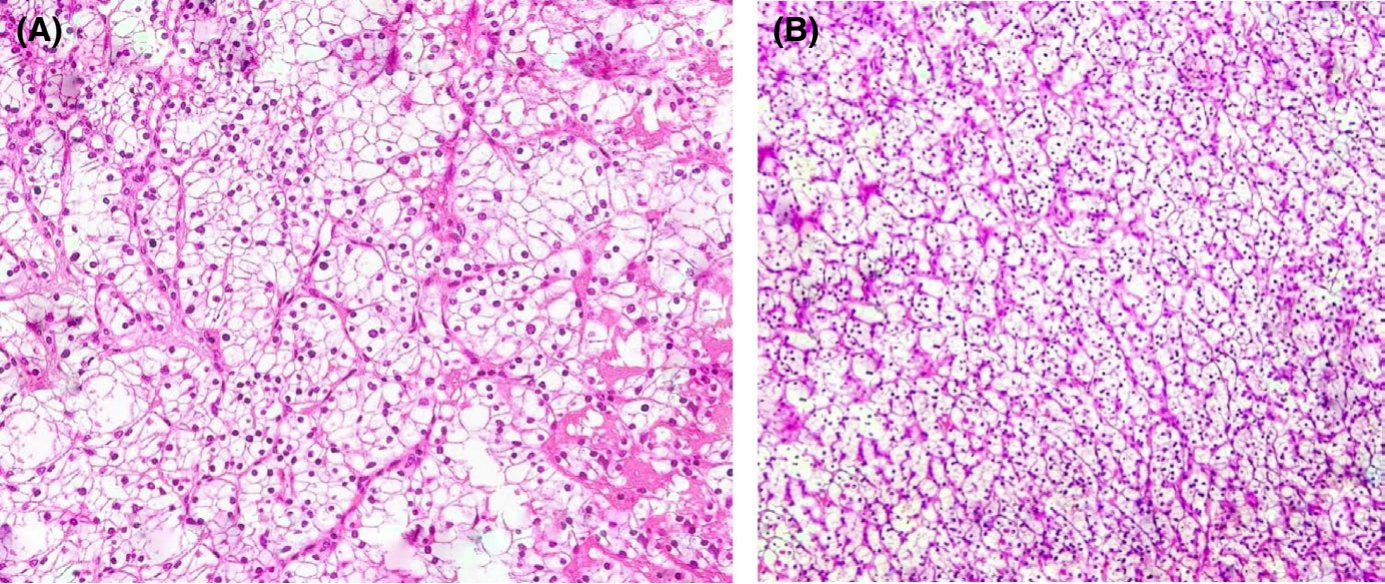
(A, B) The hematoxylin and eosin stain (H&E) of renal and bladder growth shows a similar fashion of epithelial cells with clear cytoplasm and prominent nuclei arranged in nests with intervening branching vascular tissue.

**FIGURE 4:**
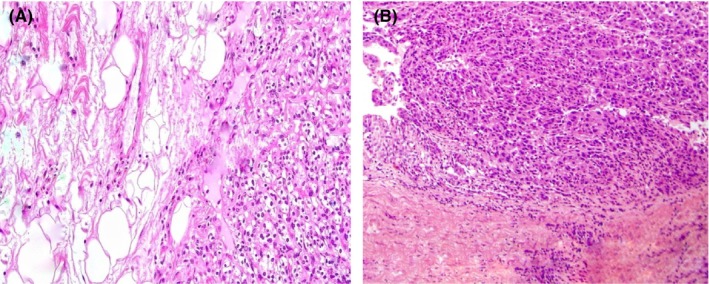
(A, B) The Hematoxylin and eosin (H&E) stain of renal tissue shows the involvement of perinephric fat and pelvicalyceal area respectively.

**FIGURE 5:**
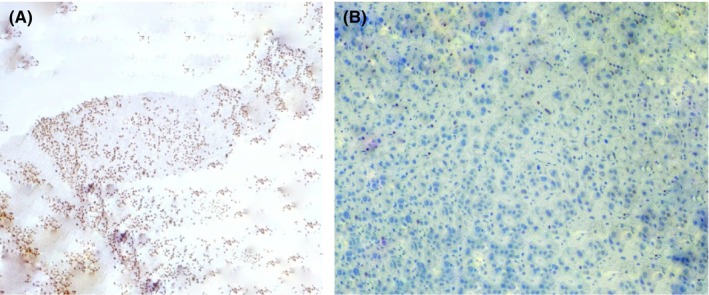
(A) Immunostaining of bladder specimen shows positivity for PAX 8 expression (200 ×) suggestive of the renal origin of the tumor. (B) Immunostaining of bladder tissue stains negative for GATA 3 expression (200 ×) suggestive of no association with urothelium.

In gross appearance, an ill‐defined hard mass pressing the hilum on one side, with detailed analysis, yielded a renal tumor that was non‐capsulated, with areas of hemorrhage and necrosis, along with cystic spaces, and depicted novel findings indicative of the clear cell carcinoma subtype of renal cell carcinoma in both tumors on microscopy (Figure 3A,B) and involvement of the perinephric and peri‐pelvic area in the renal specimen (Figure 4A,B). Microscopic examination also revealed tumor cells exhibiting clear cytoplasm, large nuclei, and prominent nucleoli, accompanied by areas of lymphocytic infiltration in the surrounding tissues. On immunohistochemical staining, the metastatic bladder tumor was PAX 8 positive with GATA 3 negative, suggestive of the renal origin of the tumor and no association with the urothelium respectively (Figure 5A,B).

### 2.5 Ongoing Follow‐Up Plan

Our patient had isolated bladder metastasis. Transurethral resection of the bladder tumor, along with radical nephrectomy, was performed at the same time for its complete resection, and the patient was given systemic therapy with tyrosine kinase inhibitors and immunotherapy agents. There was no residual or recurrent lesion detected in the urinary bladder on regular cystoscopy for 2 years, and no recurrent tumor was detected on contrast‐enhanced computed tomography of the chest, abdomen, and pelvis annually for 4 years.

## 3 Discussion

It has been reported that among all patients with Renal Cell Carcinoma (RCC), only 20%–30% experience metastasis. Metastasis to the urinary bladder in Clear Cell Renal Cell Carcinoma (ccRCC) is extremely rare, which accounts for < 2%. The literature has described such metastasis to be synchronous or metachronous after the completion of initial treatment. In contrast to this, our patient had a solitary lesion in the urinary bladder along with a primary renal tumor [6]. Such a case of metastatic ccRCC at the time of presentation has not been reported yet. The pathological evidence underlying the spread of RCC to the urinary bladder remains unclear. Several possible mechanisms and theories have been proposed, including hematogenous, lymphatic, urinary spread, as well as drop metastasis [7]. The hematogenous spread occurs through general circulation involving renal veins, the inferior vena cava, or periureteral veins. Involvement of the regional lymphatics can also result in distant metastasis. Urinary spread can be suspected in cases of involvement of the renal pelvis or collecting duct. There is also another possibility of direct intraluminal transit of tumor cells within the distal urothelium resulting in metastasis to pelvic organs, including the urinary bladder. Although in our case, neither was there any renal vessel invasion nor regional lymphadenopathy, and the primary tumor invaded the renal pelvis, the pathogenesis remains inconclusive [8, 10, 14].

Renal cell carcinoma shares differentials with many benign pathologies like renal angiomyolipoma, oncocytoma, renal cysts, and pyelonephritis [15].

Treatments for the metastatic ccRCC are limited owing to poor treatment response to chemotherapy, radiation, and immune therapy [3]. In metastatic renal carcinoma, the 5‐year survival rate is 0%–20%, while in patients who have tumor constraints to the kidney, survival is 95% [16]. The prognosis is good when only a single metastatic lesion is present within the urinary bladder because additional systemic treatment is not needed after the surgical removal of the metastatic lesion. In 20%–30% of patients diagnosed with metastasis, clinicians use memorial Sloan Kettering cancer center (MSKCC) and IMDC (international metastatic renal ca database consortium) to asses prognosis and guide treatment. The patients are stratified into 3 prognostic categories based on the number of risk factors: favorable, intermediate, and poor [17]. Tyrosine kinase inhibitors (TKIs) are utilized as a systemic therapy in metastatic renal cell cancers, with or without combination with immunotherapies. TKI monotherapy with sunitinib has been evaluated in a randomized trial compared with interferon alpha across all risk groups, and it was observed that median progression‐free survival is longer with sunitinib than with IFN alpha across all risk groups [18]. In another study, TKI and immunotherapy combinations were utilized as first‐line treatment in metastatic renal cell cancer. Nivolumab and cabozantinib were compared against sunitinib and showed superior overall survival in comparison to sunitinib alone [19]. Similarly, in another study, patients with a single metastatic site were compared with those with multiple metastatic sites, all of whom were given tyrosine kinase inhibitors. It was found that patients with a single metastatic site had longer overall survival (not reached vs. 66 months) and progression‐free survival (37 vs. 17 months) [20]. Hence, for patients who are candidates for systemic treatment, first‐line management is challenging, and clinicians can take help from evaluating IMDC, MSKCC risk groups, comorbidities, the number and locations of metastasis, and tumor histology in the treatment decision.

Since our patient had isolated bladder metastasis, transurethral resection of the bladder tumor along with radical nephrectomy was performed at the same time for its complete resection, and the patient was kept on a sunitinib and nivolumab combination for about 4 years.

## 4 Conclusion and Results

The literature includes numerous reports of the clear cell carcinoma subtype of renal carcinoma with synchronous and metachronous bladder metastases; however, the concurrent presence and management of a primary tumor alongside a metastatic bladder lesion represent a novel, rare, and challenging clinical scenario. Uro‐oncologists must be aware of the potential for isolated bladder lesions in patients with clear cell carcinoma at the time of presentation. This awareness will facilitate timely consideration of appropriate management strategies to address the dual existence of these tumors effectively.

## Author Contributions

**Rao Nouman Ali:** conceptualization, project administration, supervision, validation, visualization, writing – original draft, writing – review and editing. **Adeel Anwaar:** conceptualization, project administration, supervision, validation, visualization, writing – original draft, writing – review and editing. **Wajiha Irfan:** conceptualization, project administration, supervision, validation, visualization, writing – original draft, writing – review and editing. **Farooq Hameed:** validation, visualization, writing – original draft. **Inam Ul Haq:** validation, visualization, writing – original draft. **Muhammad Asif Raza:** project administration, supervision, validation, visualization, writing – original draft. **Riyan Imtiaz Karamat:** data curation, visualization, writing – original draft, writing – review and editing. **Aymar Akilimali:** validation, visualization, writing – original draft.

## Ethics Statement

This is a case report utilizing anonymized patient information and so was classified as exempt from review by the Institutional Review Board.

## Consent

A written informed consent was obtained from the patient based on the journal's policies.

## Conflicts of Interest

The authors declare no conflicts of interest.

## Data Availability

Data can be requested from corresponding author.
